# Lymph node ratio is an independent prognostic factor for patients after resection of pancreatic cancer

**DOI:** 10.1186/s12957-015-0510-0

**Published:** 2015-03-13

**Authors:** Han-xiang Zhan, Jian-wei Xu, Lei Wang, Guang-yong Zhang, San-yuan Hu

**Affiliations:** Department of General Surgery, Qilu hospital, Shandong University, No. 107, Wenhua West Road, Lixia District, Jinan, Shandong Province 250012 China

**Keywords:** Pancreatic cancer, Lymph node ratio, Prognosis, Surgery

## Abstract

**Background:**

The prognostic value of lymph node ratio (LNR) in pancreatic cancer remains controversial. In the current retrospective study, we assessed the value of LNR on predicting the survival of postoperative patients with pancreatic cancer.

**Methods:**

Medical records of patients who underwent pancreatic resection for pancreatic cancer in the department of general surgery, Qilu Hospital, Shandong University were reviewed retrospectively. Demographic, clinicopathological, tumor-specific data, and histopathological reports were collected. Univariate and multivariate survival analyses were performed.

**Results:**

A total of 83 patients with pancreatic cancer were collected. The mean number of examined LN was 8.2 ± 6.1 (0 to 26). Differential degree (low) (*P* = 0.019, hazard ratio (HR) = 2.276, 95% confidence interval (CI): 1.171 to 4.424) and LNR >0.2 (*P* = 0.018, HR = 2.685, 95% CI: 1.253 to 5.756) were independent adverse prognostic factors according to the multivariate survival analysis.

**Conclusions:**

Our study indicated that LNR >0.2 was an independent adverse prognostic factor for pancreatic cancer, which may provide important information for prognostic assessment.

## Background

Pancreatic cancer is the fourth most frequent cause of cancer death in the United States with an overall 5-year survival rate of 5% [1]. Only 20% of cases can be resected when diagnosed. However, the 5-year survival rate is reported to range only between 15% and 25% [2].

Several factors are related to the prognostic outcome of patients with resection of pancreatic cancer, including tumor stage, histologic differentiation, tumor size, lymph node (LN) status, and resection margin status [3]. Of them, the prognostic value of LN status is always controversial. Several studies have demonstrated that LN metastasis is associated with poor prognosis of patients with pancreatic cancer, whereas some other studies have not observed the associations between LN metastasis and survival outcomes [4].

To assess the prognostic value of LN involvement better, the importance of lymph node ratio (LNR) has been highlighted, which is determined by dividing the total number of metastatic LNs by the total number of examined LNs [5]. Many studies have identified that LNR is a valuable prognostic factor in pancreatic cancer patients [5]. Nevertheless, its prognostic value in node-positive patients has not been shown in other studies [4]. Additionally, the cutoff values of LNR are inconsistent in different studies. LNR ≥0.2, 0.15, and 0.1 have all been reported as an independent poor predictive factor [6-9].

The current study aimed to assess the prognostic value of LNR in postoperative patients with pancreatic cancer.

## Methods

This study has been approved by the ethics committee on scientific research of Shandong University, Qilu Hospital and has been performed in accordance with the ethical standards and according to the Declaration of Helsinki. Written informed consent was obtained from all subjects.

A series of 83 patients who underwent resection for pancreatic cancer in the department of general surgery, Qilu Hospital, Shandong University was collected for analysis. Surgical procedures were conducted by senior surgeons. Medical records were reviewed retrospectively, and demographic, clinicopathological, tumor-specific data, and histopathological reports were collected. TNM staging was defined according to the National Comprehensive Cancer Network (NCCN) Clinical Practice Guidelines in Oncology (NCCN Guidelines®) Pancreatic Adenocarcinoma Version 1, 2014 (http://www.nccn.org/professionals/physician_gls/f_guidelines.asp). Follow-up data were acquired from hospital records supplemented with telephone contact. The end point was overall survival. Survival time was calculated according to the date of death or as the time between the last follow-up date and the operation date.

Statistical analysis was conducted using SPSS v.13.0 software (SPSS Inc., Chicago, IL, USA). A value of *P* < 0.05 was considered as statistically significant. Graphs were produced by GraphPad Prism 5 Software (GraphPad, San Diego, CA, USA). The Kaplan-Meier method and Cox regression were used for univariate and multivariate survival analyses, respectively.

## Results

### The clinicopathologic characteristics of patients

A total of 83 patients with pancreatic cancer were collected in the current study, including 53 males and 30 females, with an average age of 61.7 ± 10.7 (range 36 to 85) years old. The average size of the tumor diameter was 4.6 ± 2.23 cm (range 0.5 to 14 cm). The total number of examined LNs was range from 0 to 26, with a mean of 8.2 ± 6.1. The average follow-up was 26.9 months (median 15, range 1 to 87 months).

### Univariate and multivariate survival analyses

**Figure 1:**
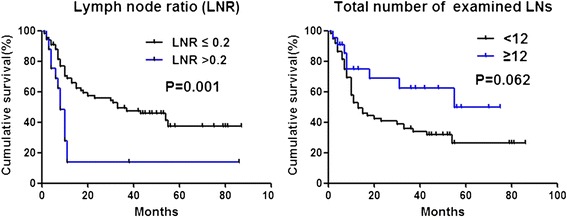
**Kaplan-Meier survival curves.** Left panel, survival analysis based on LNR. Right panel, survival analysis based on the total number of examined LN. LN, lymph node; LNR, lymph node ratio.

The median survival was 20 months. The overall 1- and 3-year survival rates were 58.6% and 42.7%, respectively. Univariate survival analysis indicated that sex, differential degree, LN staging, TNM staging, LNR, and total number of examined LNs were potential prognostic factors (Table 1, Figure 1). Multivariate analysis demonstrated that differential degree (low) and LNR >0.2 were independent adverse prognostic factors (*P* = 0.019, hazard ratio (HR) = 2.276, 95% confidence interval (CI): 1.171 to 4.424; *P* = 0.018, HR = 2.685, 95% CI: 1.253 to 5.756) (Table 2).

**Table 1:** **Univariate analysis of factors predictive of poor overall survival**

**Variables**	**Case number (** ***n*** **)**	**Univariate analysis**		
		**Overall survival (median ± SE, months)**	**1-year survival rate (%)**	***P*** **value**
Sex				0.041
Male	53	15 ± 3.4	54.5	
Female	30	54 ± 11.0	65.9	
Age(years old)				0.718
<65	50	20 ± 9.6	62.0	
≥65	33	33 ± 15.9	53.2	
Diabetes				0.168
No	74	23 ± 10.8	61.9	
Yes	9	10 ± 1.5	33.3	
Locations				0.353
Head	52	31 ± 14.8	63.1	
Body-tail	31	15 ± 4.7	51.6	
Tumor size (cm)				0.606
≤3	12	20 ± 7.4	63.6	
>3	70	23 ± 11.0	57.3	
Differential degree^a^				0.039
High/moderate	55	33 ± 12.8	66.3	
Low	22	10 ± 1.4	40.8	
Tumor staging				0.664
T1/T2	69	18 ± 9.6	57.3	
T3/T4	14	23 ± 13.7	64.3	
Lymph node staging				0.041
N0	53	33 ± 14.1	69.0	
N1	30	11 ± 1.3	39.7	
TNM staging				0.014
I/II	41	54 ± 15.5	72.1	
III/IV	42	11 ± 3.1	45.5	
Perineuronal invasion				0.082
No	48	43 ± 22.8	69.4	
Yes	35	11 ± 1.0	44.4	
LNR				0.001
≤0.2	66	33 ± 13.6	69.0	
>0.2	17	8 ± 1.1	13.8	
Total number of examined LNs				0.062
<12	61	15 ± 4.3	53.4	
≥12	22	55 ± None^b^	74.9	

^a^The differential degree of six cases is not recorded. ^b^None: if the number of censored data is more than 50% of the total, median survivals cannot be calculated by SPSS. LN, lymph node; SE, standard error.

**Table 2:** **Multivariate analysis of factors predictive of poor overall survival**

**Variables**	**Hazard ratio**	**95% confidence interval**	***P*** **value**
LNR (>0.2)	2.685	1.253 to 5.756	0.018
Differential degree (low)	2.276	1.171 to 4.424	0.019
Sex (female)	0.578	0.298 to 1.124	0.094
Lymph node staging (N1)	0.761	0.284 to 2.042	0.717
TNM staging (III/IV)	1.5	0.772 to 2.914	0.229

LNR, lymph node ratio.

## Discussion

LN involvement remains one of the most important factors for predicating survival of patients with resection of pancreatic cancer [5,10]. However, both LN status and the numbers of examined LNs are imperfect as the sole predictor. LNR not only provides information regarding the number of positive LNs but also gives an estimate of the adequacy of LNs obtained [9], which is a significant modifier of the effect of LN status and the numbers of examined LNs on survival of patients with resected cancer [11]. LNR has been identified as a tool to predict outcome in cancers of the esophagus [12], stomach [13], colon [14], and ampulla of Vater [15]. However, the association of LNR and overall survival in pancreatic cancer has not been well defined. We showed that LNR is negatively correlated with the overall survival with a cutoff value of 0.2.

There is no consensus on the best cutoff value for LNR. Pawlik and colleagues used categories of LNR <0.2, 0.4, and >0.4 [16], while House *et al*. used 0.18 as a cutoff value [17]. Ashfaq *et al*. indicated that LNR cutoff of 0.1 was statistically significant for survival discrimination [9]. Our study demonstrated that patients with LNR >0.2 displayed poor prognosis, as reported by previous studies [6,7].

In addition, we evaluated the prognostic role of examined LNs, which indicated that total number of examined LNs ≥12 was potentially associated with improved survival. Several studies have reported the link between longer survival and total number of examined LNs [18,19]. Our study might also indicate that standard lymphadenectomy is enough, because the mean number of LNs resected in patients with pancreatic cancer who underwent standard lymphadenectomy in the randomized controlled trials (RCTs) was 13 to 17 [20-22]. Extended lymphadenectomy increases the total number of examined LNs, but there are no significant differences in the overall survival between patients who underwent pancreatic cancer surgery with extended lymphadenectomy and those who underwent operation with standard lymphadenectomy [23,24]. On the contrary, extended lymphadenectomy may increase postoperative morbidities and mortalities and decrease quality of life [24,25]. Although there was a debate on the value of extended lymphadenectomy in the past, the ideas are beginning to converge. Unnecessary extended lymphadenectomy should be avoided, which has been recommended by the NCCN Clinical Practice Guidelines in Oncology (NCCN Guidelines®) Pancreatic Adenocarcinoma Version 1, 2014.

Both the LN status and the LNR are influenced by the total LN harvested [10]. Valsangkar and colleagues analyzed 14,907 patients in a national database and 902 patients treated at a single large institution, which showed that a minimum of 13 to 16 LNs must be examined to accurately predict survival [10]. The mean number of the total examined LN was 8.2 ± 6.1 in our study, which might not do full justice to the prognostic value of LNR. Nevertheless, a moderate number (6 to 12 LNs) of the total examined LN could partly predict survival [10]. What we need to emphasize is that the retrieval of the lymph nodes not only depends on the scope of the lymphadenectomy but also depends on the seriousness of the pathologist. Only surgeons and pathologists cooperated closely may accurately evaluate the value of LNR.

## Conclusions

The present study demonstrated that LNR >0.2 was an independent adverse prognostic factor, which is powerful and useful for prognostic assessment for pancreatic cancer.

## Abbreviations

LN: lymph node
LNR: lymph node ratio
